# Efficacy and Safety of Hyperbaric Oxygen Therapy for Radiation-Induced Hemorrhagic Cystitis: A Systematic Review and Meta-Analysis

**DOI:** 10.3390/jcm13164724

**Published:** 2024-08-12

**Authors:** Teng-Kai Yang, Yu-Jen Wang, Hsing-Ju Li, Ya-Fang Yu, Kai-Wen Huang, Jason Chia-Hsien Cheng

**Affiliations:** 1 Department of Surgery, Yonghe Cardinal Tien Hospital, New Taipei City 23445, Taiwan; a9508d2@cthyh.org.tw; 2 School of Medicine, College of Medicine, Fu-Jen Catholic University, New Taipei City 242062, Taiwan; 138697@mail.fju.edu.tw; 3 Division of Radiation Oncology, Department of Oncology, National Taiwan University Hospital, Taipei 100225, Taiwan; 4 Graduate Institute of Clinical Medicine, National Taiwan University College of Medicine, Taipei 100225, Taiwan; 121446@ntuh.gov.tw (H.-J.L.); p09421405@ntu.edu.tw (Y.-F.Y.); skywing@ntuh.gov.tw (K.-W.H.); 5 Department of Surgery, National Taiwan University Hospital, Taipei 100225, Taiwan; 6 Graduate Institute of Oncology, National Taiwan University College of Medicine, Taipei 100225, Taiwan

**Keywords:** radiation-induced hemorrhagic cystitis, hyperbaric oxygen therapy, hematuria, systematic review, meta-analysis

## Abstract

**Background:** Radiation-induced hemorrhagic cystitis (RHC) is a chronic inflammatory disease in patients undergoing radiation therapy that causes a cluster of symptoms which may have a latent period of months to years. The current non-invasive treatments include drug treatment and hyperbaric oxygen therapy (HBOT), which has been widely applied for RHC so far but with limited evidence. Thus, we conducted a systematic review and meta-analysis to clarify the effects and safety of HBOT for RHC. **Methods:** A systematic review and meta-analysis were utilized, searching in the databases of Embase, Pubmed, and Web of Science. The primary endpoint of the present study was complete remission of hematuria. The meta-analysis was conducted using a random effects model, and a pooled odds ratio with 95% CI was calculated. **Results:** A total of 317 studies were searched and fourteen articles with 556 patients were collected. The results showed that a total of 500 patients (89.9%) had symptom improvement, and the pooled results demonstrated that 55% of patients with HBOT had complete remission of hematuria (95% CI 51–59%). **Conclusions:** A significant improvement of symptoms when treated with HBOT was shown in this meta-analysis for patients with RHC.

## 1. Introduction

Cancer has been the leading cause of death in Taiwan for more than four decades. The incidence of urological and gynecological pelvic cancers has risen, with a growing health burden worldwide [1]. Radiation therapy (RT) for urological and gynecological cancers, as the primary or adjuvant therapy, treats more than 150,000 patients worldwide each year [2], and radiation exposure to the pelvic organ can be followed by late urinary complications, such as radiation cystitis, urethral stricture, and fistulas, which may occur decades after RT [3]. Although new techniques in RT such as pelvic Image-Guided Radiation Therapy (IGRT) or Intensity-Modulated Radiation Therapy (IMRT) may potentially lower the range of healthy tissue damage in the treatment field, the risk of early and late complications cannot be dismissed. The late effects of RT are usually dose-responsive and affect slowly proliferating tissues, remaining major issues [4].

RT may induce vascular changes, subendothelial hyperplasia, edema, and tissue thickening, gradually reducing the blood supply to the tissue and creating a hypocellular and hypoxic environment, followed by perivascular fibrosis, ischemia, and then vascular occlusion [5]. The pathogenesis results in a cluster of lower urinary tract symptoms, such as common hematuria, urinary incontinence, dysuria, urgent urination, frequent urination, and pelvic pain, which are irritative burdens on the patient [5,6]. 

Radiation-induced hemorrhagic cystitis (RHC) is one of the late-onset complications of RT of the pelvic organ, with an incidence rate of approximately 5–10% [7]. RT with radiation doses ranging from about 45–74 Gy may cause RHC [8], and the various associated symptoms, of which refractory bleeding can lead to life-threatening hypovolemic shock and others might lead to the need for major surgery, such as urinary diversion and cystectomy, from which the mortality rate can be as high as 44% [9].

The non-invasive managements for RHC include systemic medical therapy, intravesical instillation, laser or electric cauterization, and hyperbaric oxygen therapy (HBOT) [8,10]. Systemic medical therapies, such as sodium pentosan polysulfate, tranexamic acid, and hyaluronic acid, are appealing due to their non-invasiveness and outpatient treatment [11], but their limitations are the time to effect and risk of thromboembolic events [8]. HBOT treats patients with 100% oxygen through inhalation while inside a pressurized treatment chamber with a dose-dependent effect, and the treatment depth and duration are crucial. HBOT can either be the primary treatment or used as an adjuvant therapy to medications or surgical techniques, and results in a benefit for wound healing and reduction in pain following pelvic irradiation [12]. However, HBOT has been associated with some adverse events, including temporary visual acuity reduction and ear compression barotrauma [13]. Currently, studies for the treatment effect of HBOT for RHC have limited evidence. Herein, we conducted a systematic review and meta-analysis to clarify the effects of HBOT for RHC. 

## 2. Methods

### 2.1. Data Sources and Literature Search Strategy

**Figure 1:**
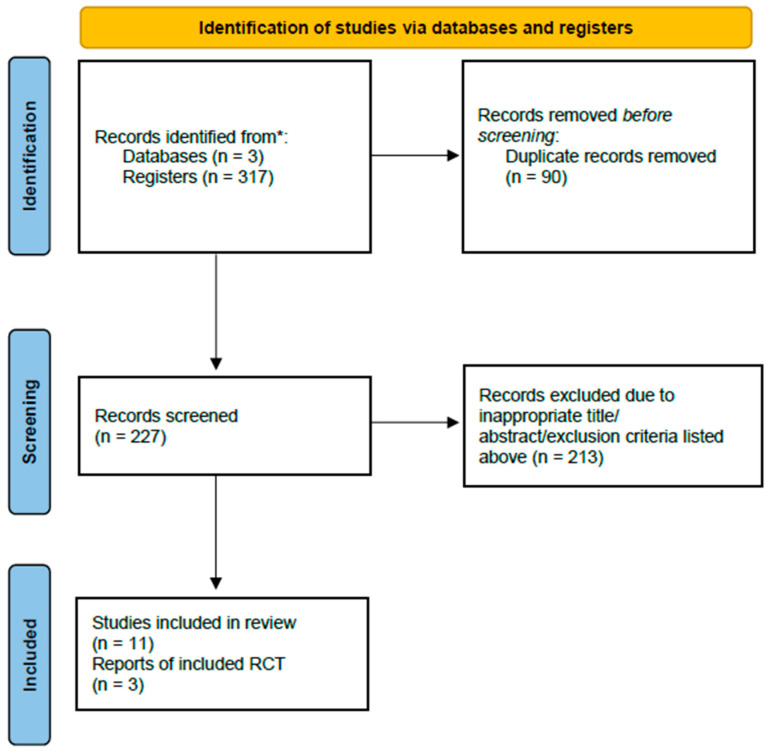
PRISMA flow diagram showing screening and selection process [14]. * The initial search identified 317 published (registered) articles from online databases including PubMed, EMBASE and Web of Science.

The present study followed the PRISMA 2020 statement, including a checklist and flow diagram (Figure 1) [14], and a registration statement was completed in the international prospective register of systematic reviews (PROSPERO). All authors were involved to screen and retrieve the studies, which were included utilizing PICOS criteria: (1) Patients: men/women with RHC; (2) Intervention: treatment with HBOT; (3) Control group: subjects receiving standard medical treatment; (4) Study design: original studies including randomized controlled trials, prospective studies, and retrospective studies. 

The searched databases, including PubMed, EMBASE, Cochrane Library, and Web of Science, were searched for eligible studies from January 2022 to March 2024. The search terms used for the diagnosis included “Hemorrhagic radiation cystitis” OR “Radiation cystitis”. The terms used to define the intervention included “Hyperbaric oxygen therapy” OR “HBOT” OR “HBO”. In addition, we also checked the reference lists of all relevant articles to identify additional studies.

### 2.2. Study Selection

The primary outcome was complete remission (CR), which was defined as the disappearance of all symptoms, including macroscopic hematuria, dysuria, etc. The reduction of the frequency or severity of macroscopic hematuria, or an improvement in at least one of the EPIC/LENT-SOMA sub-scores with statistical significance, compared to the standard treatment group, was defined as partial remission (PR).

Publications of randomized controlled trials (RCTs) and prospective and retrospective studies with primary outcomes of CR and PR in patients with RHC treated with HBOT were included. The exclusion criteria were previous definitive therapy for cancer in non-pelvic areas, radiation cystitis patients not treated with HBOT, and non-English language studies. 

HBOT was administered to all patients in a multiplace or monoplace compression chamber with 100% oxygen for at least 60 min and with a standardized pressure between 1.8 and 2.5 atmospheres. The number of sessions ranged between 40 and 120, with a follow-up duration of 6 to 12 months or longer follow.

### 2.3. Data Extraction and Quality Assessment

**Figure 2:**
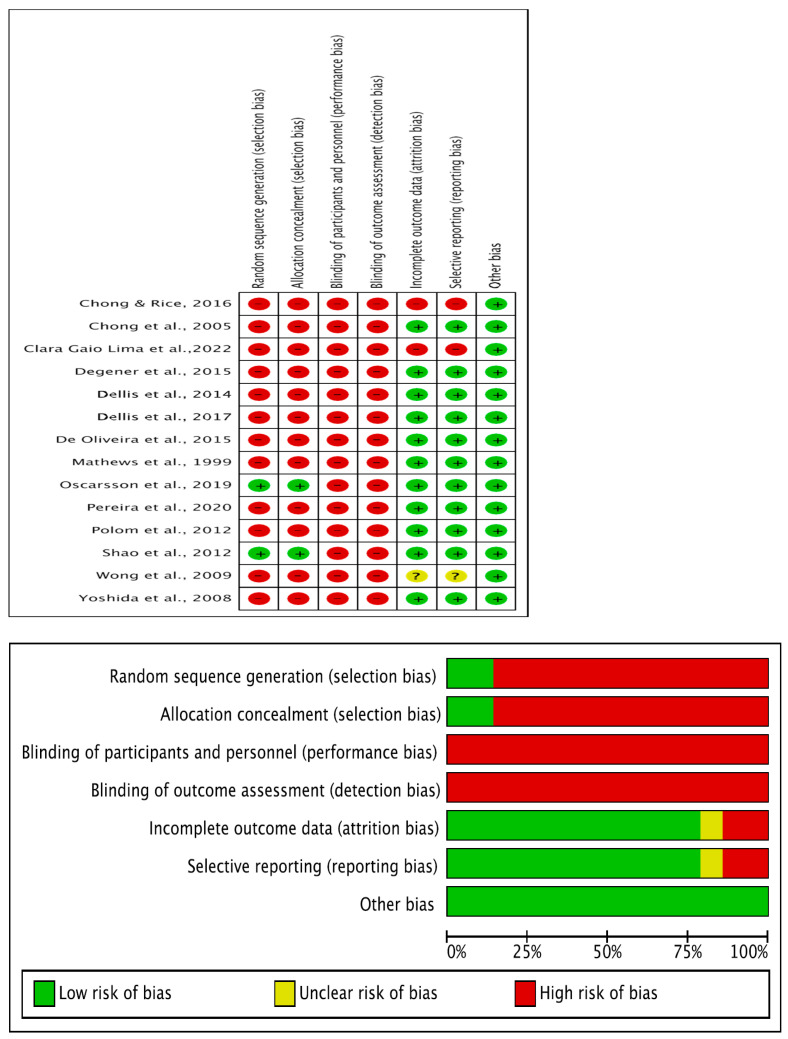
Risk of bias graph: a review of authors’ judgements about each risk of bias item presented as percentages across all included studies [16,17,18,19,20,21,22,23,24,25,26,27,28,29].

The following information was extracted for each eligible study: title of the article, first author, publication year, trial phase, study design, applied agents, sample size, and national clinical trial (NCT) identification number. The risk of bias for individual studies was assessed at the study level based on the Cochrane Collaboration’s tool for randomized trials, which included the following domains: random sequence generation, allocation concealment, blinding, incomplete outcome data, and selective outcome reporting (Figure 2) [15]. The evaluation of the risk of bias was conducted by the Review Manager (RevMan, V.5.4.1, Nordic Cochrane Centre, Cochrane, Copenhagen, Denmark).

### 2.4. Statistical Analysis

We calculated the pooled odds ratio (OR) and 95% confidence interval (CI) for CR, the primary outcome, for which *p* < 0.05 was considered statistically significant. Heterogeneity among studies was quantified by the I-squared (I^2^) test, and I^2^ > 50% was considered substantial heterogeneity. Due to a high percentage of retrospective studies possibly being included, the meta-analysis was conducted using the random effects model under the assumption of significant heterogeneity. The statistical analysis was conducted using Review Manager (RevMan5.4.1).

## 3. Results

### 3.1. Study Selection and Characteristics of the Included Studies

The initial search identified 317 articles from online databases. After the screening process, duplicates (90) and irrelevant studies (213) were excluded. Finally, 14 articles were included in this meta-analysis (Figure 1). Three were RCTs [16,17,18], three were prospective studies [19,20,21], and eight were retrospective studies [22,23,24,25,26,27,28,29]. A total of 556 patients were included in the meta-analysis. The mean age of included patients was 67 years, with a range from 15 to 86 years. The total sessions of HBOT were between 40–120.

### 3.2. Risk of Bias

Three domains of the included studies were found to have a low risk of bias (incomplete outcome data, selective outcome reporting, and other bias) and four domains with high risk of bias (random sequence generation, allocation concealment, blinding of participants and personnel, and blinding of outcome assessment) (Figure 2). The summary showed that there were relatively high levels of risk for selection bias, performance bias, and detection bias, which may be due to the higher percentage of retrospective studies in the present meta-analysis.

### 3.3. Major Outcomes: Complete Remission and Partial Remission

**Figure 3:**
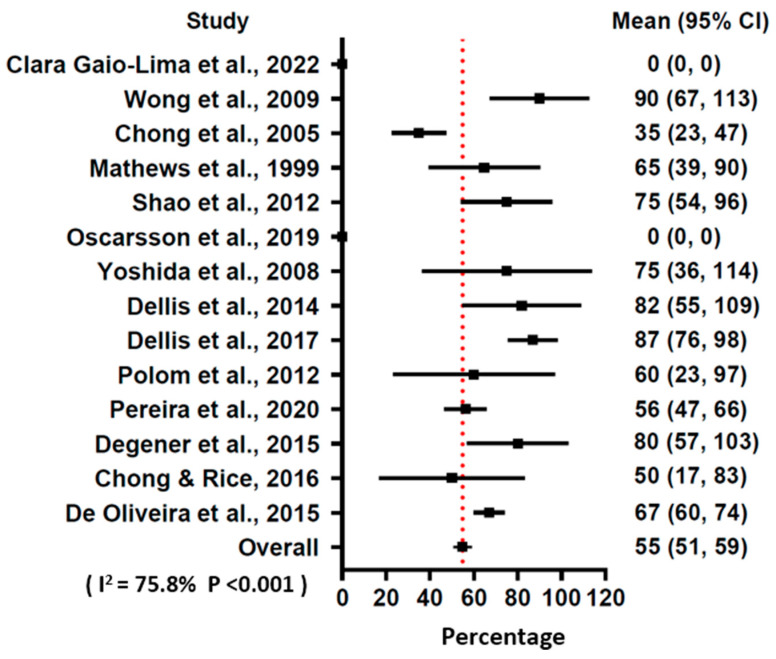
Forest plot of hazard ratio of complete remission using a random effects model [16,17,18,19,20,21,22,23,24,25,26,27,28,29].

**Figure 4:**
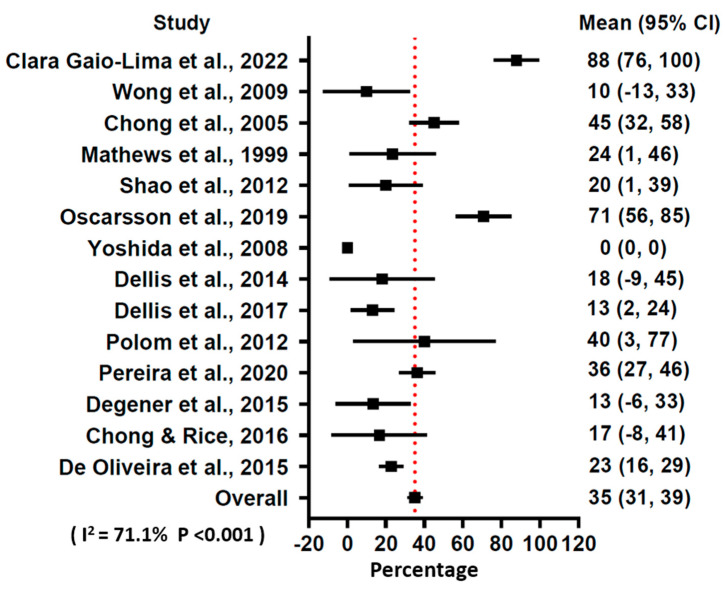
Forest plot of hazard ratio of partial remission using a random effects model [16,17,18,19,20,21,22,23,24,25,26,27,28,29].

Based on the results of the studies, a total of 500 (89.9%) patients had an improvement of symptoms after 14–120 treatment sessions. Among them, 305 patients had CR and 195 had PR of RHC symptoms (Table 1). The pooled results of the meta-analysis showed that HBOT had a 54.9% (prediction interval 51–59%) rate of CR of the symptoms for the patients with RHC (Figure 3) and a 35.1% (prediction interval 31–39%) rate of PR of symptoms (Figure 4). The I square of the forest plots for CR and PR were 75.8% and 71.1% respectively, indicating the substantial heterogeneity of the present study. The I^2^ ratio in the forest plot represented the variance in true effects rather than sampling error and could be corrected with random effects assumption. A recent study utilized the prediction interval to quantify heterogeneity in a meta-analysis, which may be another optimal tool to quantify heterogeneity [30]. 

### 3.4. Treatment-Related Adverse Events

**Figure 5:**
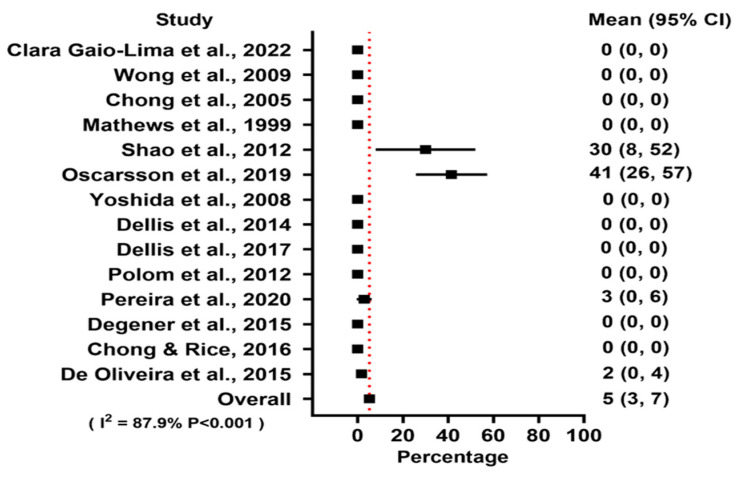
Forest plot of adverse events using a random effects model [16,17,18,19,20,21,22,23,24,25,26,27,28,29].

The stopping criteria for oxygen administration is the development of serious side effects of HBOT, such as claustrophobia, fever, vomit, more than one oxygen-induced episode of generalized seizures, pneumothorax, pulmonary barotrauma, or pronounced deterioration of cardiovascular or respiratory failure. The forest plot for treatment-related adverse events is shown in Figure 5. No major adverse events were recorded for all included studies, and the mean minor adverse event rate was 5.2%. 

For one included RCT, 17 (41%) of 41 patients in the HBOT group experienced occasional grade 1–2 adverse events, related to sight and hearing, during the period of HBOT [17]. Another included RCT showed that the main side effect of hyaluronic acid instillation was urinary tract infection due to repeated urethral catheterization. The incidence seemed higher than HBOT (42.8% vs. 10%, *p* = 0.034) in the first 6 months but showed no significant difference at 12 months (50% vs. 25%, *p* = 0.13) and 18 months (50% vs. 30%, *p* = 0.14) after treatment [16].

## 4. Discussion

The present study showed that HBOT significantly improved RHC-related symptoms and the quality of life of patients with RHC. One previous meta-analysis with 499 patients and a median age of 68 years showed that 84% of them had recovery of hematuria [5]. As a comparison, our study had an acceptable CR rate of 54.9% (prediction interval 51–59%) and PR rate of 35.1% (prediction interval 31–39%). Another systematic review showed that from 20 obtained studies, the weighted average rates overall and for CR were 87.3% and 65.3%, respectively, for RHC patients who received HBOT [31], which were comparable with the present study. One included RCT in the present study compared the effects of intravesical hyaluronic acid with HBOT and showed similar sustained amelioration of hematuria of radiation cystitis between the two treatments. A significant improvement of visual analogue scale (VAS) was achieved in both groups. Despite the slightly higher incidence of urinary tract infection, the intravesical instillation of hyaluronic acid was cheaper and much more convenient than HBOT [16]. Another included RCT compared HBOT with standard care and demonstrated that HBOT had a significant improvement on Expanded Prostate Cancer Index Composite (EPIC) scores and 36-item Short Form (SF-36) responses. Further, other included studies showed remarkable responses to HBOT for patients with RHC. 

The effect of HBOT on health-related quality of life (HRQOL) has been investigated in a few of the included studies. Oscarsson et al. found that HRQOL score improved in the HBOT group in four domains, including physical and individual quality of life aspects (physical functioning, energy, bodily pain, and general health); but the magnitudes of these improvements were small and without statistical significance, and a longer time may be needed for the social and psychological improvements to become evident [17]. Another study with a small patient number, using of the generic SF-36 questionnaire, described a statistically significant improvement only in the role and emotional domains for patients with RHC [32]. 

Some potential therapeutic approaches such as antifibrotic or antioxidant pharmacological agents may have therapeutic effects. Vitamin E plays an important role in preventing lipid peroxidation in the cellular membrane and can protect cells from oxidative stress, and a clinical trial of RT patients treated with a PTX/vitamin E combination showed a significant improvement in RT-induced fibrosis [6]. Edaravone, another potential treatment option for RHC, is a low-molecular-weight free-radical scavenger that suppressed oxidative stress, protected against bladder dysfunction, and reduced cyclophosphamide-induced hemorrhagic cystitis in a rat model [33]. All of the above potential treatments needed more evidence to confirm their therapeutic effects.

The lack of consistency in the evaluation tools for the therapeutic effects on hematuria across all studies deserved attention. The assessment tools used in the other meta-analysis consisted of a patient-based description of symptoms, the Common Terminology Criteria for Adverse Events, and RTOG/EORTC and VAS scores. The assessment tools in our study included LENT-SOMA, RTOG/EORTC, macroscopic hematuria, EPIC, SF-36, and VAS. This diversity of tools may limit the strength of the recommendations from this meta-analysis. In addition, the number of studies searched was relatively small, which may compromise the interpretations of the included studies and included patients. 

Another study mentioned the importance of the interval between each RT session, the onset of hematuria, and the start of HBOT. A possible explanation for the negative effect of a long interval between the onset of hematuria and start of HBOT is that the early application of HBOT in the process of RHC may be likely to be more effective in tissue regeneration, and then interrupt the vicious circle of bladder tissue damage due to chronic hypoxia [27]. In addition, higher radiation doses may cause a higher severity of RHC due to the short interval between the completion of RT and the onset of hematuria. The resulting hematuria may happen earlier during the disease process and may be more difficult to resolve with HBOT.

One important issue for radiation cystitis is that it can occur many years after RT. Effective treatment remains a highly relevant clinical issue, even with ongoing technical improvements in the field of RT [13]. Although there were many reports with the improved outcomes by HBOT for radiation cystitis, most of them were small single-center studies. The solid evidence from prospective studies is still lacking. Moreover, the pathophysiological changes induced by radiation evolve over time and the dose and timing of HBOT might also be important for the individual response to the treatment [16]. These issues are all needed to be investigated in further research.

There are some limitations in our investigation for this meta-analysis. First, there were few prospective studies or RCTs and a relatively small number of patients to be analyzed with satisfactory statistical power. The higher percentage of retrospective studies lead to higher levels of risk for selection bias, performance bias, and detection bias. Second, there were different alternative treatments, including intravesical hyaluronic acid or laser coagulation, used in the control groups in the included studies. The various measurements and evaluation tools were inconsistent between the included studies, which were not only challenging when comparing the main outcomes for all studies, but also caused heterogeneity and difficulty in conducting the meta-analysis. Third, the different time points for the endpoint evaluations between studies and the various severities and gradings of RHC might be other confounding factors in the included studies. Nevertheless, the present study showed that HBOT significantly improved hematuria and the quality of life of patients with RHC. 

Despite the above limitations, this meta-analysis showed that HBOT is effective in the treatment of hematuria and other symptoms related to RHC. Factors that could be associated with suboptimal outcomes or failure of HBOT include the need for blood transfusions before HBOT, the use of anticoagulant therapy, the number of HBOT sessions, the interval between the onset of hematuria and initiation of HBOT, and the total radiation dose [31]. More prospective studies are needed to address the benefits and cost effectiveness of HBOT in the future.

## 5. Conclusions

In conclusion, our results showed that HBOT showed significant improvements for the symptoms of RHC. Treatment with HBOT is well tolerated, with very rare occurrence of severe and irreversible adverse effects.

Since novel radiation modalities are emerging promisingly, the effects of HBOT for RHC patients will become more apparent in the future, as symptoms can develop for a long time after RT. Since the overall quality of evidence of the studies included in the present study is not adequate, and as HBOT requires commitment from patients and was not available as an insurance-covered healthcare resource, more prospective randomized controlled trials with longer follow up durations and cost-effectiveness analysis will answer current and incoming questions.

## Figures and Tables

**Table 1:** Baseline characteristics and results of the included studies.

Study Name	Study Design	Pts	Median Age			Sex M/F	Management Tools	Outcome		
				Median Follow Up	Median Sessions			CR (%)	PR (%)	AE (%)
				Months	(Range)					
Mathews et al., 1999 [18]	RCT	17	62	21	14		Macroscopic hematuria	11 (64.7)	4 (23.5)	0
Shao et al., 2012 [16]	RCT	20	60.3	18.0	30	12/8	VAS scale	15 (75.0)	4 (20.0)	6 (30%)
Oscarsson et al., 2019 [17]	RCT	41	64.0	7.8	(30–40)	29/12	EPIC SF-36	-	29 (70.7)	17 (41%)
Yoshida et al., 2008 [21]	Prospective	8	64.3	15.5	19 (10–42)	5/3	Macroscopic hematuria	6 (75.0)	0	0
Dellis et al., 2014 [19]	Prospective	11	72	17.8	32.8 (27–44)	10/1	RTOG/EORTC	9 (81.8)	2 (18.2)	0
Dellis et al., 2017 [20]	Prospective	38	70	29.3	33 (20–78)	33/5	RTOG/EORTC	33 (86.8)	5 (13.2)	0
Polom et al., 2012 [22]	Retrospective	10	68.9	24.5	43.4 (8–60)	7/3	Macroscopic hematuria	6 (60.0)	4(40.0)	0
Pereira et al., 2020 [23]	Retrospective	105	65	63.0	40 (10–110)	54/51	LENT-SOMA	59 (56.2)	38 (36.2)	3 (2.9%)
Degener et al., 2015 [24]	Retrospective	15	71	68.0	34	12/3	RTOG/EORTC	12 (80.0)	2 (13.3)	0
Chong and Rice, 2016 [25]	Retrospective	12	78	14.8		-	Macroscopic hematuria	6 (50.0)	2 (16.7)	---
De Oliveira et al., 2015 [26]	Retrospective	176	61.9	12	36.5 (7–179)	65 /111	Macroscopic hematuria	118 (67.0)	40 (22.7)	3 (1.7%)
Chong et al., 2005 [27]	Retrospective	60	70	6	33 (9–63)	55/5	Macroscopic hematuria	21 (35.0)	27 (45.0)	---
Wong et al., 2009 (poster) [28]	Retrospective	10	42.7		26.4 (10–40)	8/2	Macroscopic hematuria	9 (90.0)	9 (10.0)	0
Clara Gaio-Lima et al., 2022 [29]	Retrospective	33	69	62	42 (18–114)	20/13	LENT-SOMA		29 (87.8)	---
TOTAL		556				310/ 217		305 (54.9)	195 (35.1)	29 (5.2%)

CR: complete remission, PR: partial remission, AE: adverse events.
